# Supplementary material for: Can Swallowing Cerebral Neurophysiology Be Evaluated during Ecological Food Intake Conditions? A Systematic Literature Review
Source: J Clin Med. 2022 Sep 18;11(18):5480. doi: 10.3390/jcm11185480 (PMC9505443; doi:10.3390/jcm11185480)
Supplement: Supplementary file 1 [file jcm-11-05480-s001.zip › jcm-1832558-supplementary.pdf]

Supplementary Table S1: Technical characteristics of EEG, fNIRS, MEG: methodology, swallowing sequences and measure of interest as reported in the included studies, compared to the fMRI gold standard.

Technique and signal type	Standard number of captors	Spatial resolution	Temporal sampling rate	Studied phase of swallowing act	Number of studies	Measures	Type of analyses [a]	Median time window from swallowing act onset (ms) [Min:Max]		Median ISI [Min:Max]	Task iterations (Min-Max)	Region of interest[c]
								Median start time	Median end time			
**EEG**<br><br>Whole head cerebral electrical activity	Whole head 64 electrodes channels	1mm	200Hz-500Hz	Preparation	3	Negative potentials [12,13,15] MRCP, BP, CNV	Topographic	-5,000<br>[-6,000 : -3,000]	1,000<br>[1,000 : 2000]	10-30s	50-480	Cz, Fz, FCz, Pz,
					1	Signal complexity through Approximate entropy[20]	Topographic	-1,024	0	30s	20	P3, P4, C3, C4, F4, T5, T5
				Preparation and execution	1	Negative potentials (MRCP) and positive potential[14]	Topographic	-1,500	1,000	10s	50	C3, C4,Cz
					4	Time-frequency based synchrony[16-18,21]	Network micro-architecture	-	-	2-3s	5	Whole head
					1	Time-frequency based magnitude[22]	Topographic	-	-	-	1	C3, C4
				Execution	1	Time frequency spectral power density [19]	Topographic	-1,000	3,000	2s	80	C3, C4,Cz
**fNIRS**<br><br>Targetted optical hemoglobins concentrations HDR	16 to 32 optodes (half emitters, half detectors)	2-3cm	7-50Hz<br><br>But the HDR has a temporal resolution >1s	Execution	6	OxyHb relative concentration alone[47–50,52,54]	Topographic	-5,000<br>[-20,000:-5,000]	37,500<br>[22,000 : 60,000]	30s [20:60s]	3-30	Caudal pericentral Cx<br>PMC, SMA, PFC
					5	OxyHb and DeoxyHb relative concentration [44-46,51,53]	Topographic	-5,000<br>[-5,000 : -5,000]	25,000<br>[15,000 : 25,000]	30s<br>[30:30s]	10-20	Inferior frontal gyrus
**MEG**<br><br>Whole head cerebral electro-magnetic activity	Whole head 275 SQUID sensors (151 to 306)	1mm	400-600Hz	Preparation	2	Equivalent magnetic dipole localization [24,27]	Tomographic	-2,500<br>[-2,500 : -2,500]	500<br>[500 : 500]	30s	30-50	**Whole head**<br>Cingulate gyrus<br>SMA<br>Insula<br>Inferior frontal gyrus
				Preparation and execution	16	Time-frequency domain ERS and ERD [25,29–39,40–43]	Tomographic	-3,000<br>[-4,000 : -400]	2,000<br>[600 : 2,000]	10-15s	40-100	**Whole head**<br>Pericentral Cx<br>PMC, SMA, PFC<br>Parietal Cx<br>Insula
				Execution	1	Averaged time-series[23]	Topographic	-500	1,500	10s	100	**Whole head :** No result
					2	Time-frequency domain ERS and ERD[26,28]	Tomographic	0	4,000<br>[3,000:5,000]	6-15s	20-100	**Whole head**<br>Pericentral Cx<br>Parietal Cx
**fMRI**[b]<br><br>Whole head BOLD signal HDR	BOLD signal	3-5mm	14.5Hz<br><br>But the HDR has a temporal resolution >1s	Preparation and execution	-	-	Tomographic	-	-	15s	10	Primary sensorimotor Cx, PMC, SMA, PFC, Heschl's gyrus, cingulate gurus, insula, Broca's areas, superior temporal gyrus, precuneus

BP : Bereitschaftspotential
BOLD : Blood oxygen level dependent
CNV : Contingent negative variation
Cx : Cortex
HDR : Hemodynamic response
ISI : Inter-stimulus interval
MRCP : Movement related cortical potential
PFC : Prefrontal cortex
PMC : Premotor cortex
SMA : Supplementary motor area
SQUID : superconducting quantum interference devices

a. A topographic analysis is limited to the superficial cortical layer, whereas a tomographic method can explore deeper structure (e.g. grey nuclei, insula, cingulum).
b. fMRI data as reported in reviews by Malandraki et al. [57] and Ludlow et al.[1,4].
c. The region of interest is defined based on the technique data reported in the included studies. Only the most relevant electrode/channels/areas across studies were reported. For MEG studies, the whole head coverage was reported as necessary to calculate the source of the activity.
For details and equivalence between the 10-10 system, Brodmann areas and gyrus, see supplementary tables 2 to 4.

Supplementary Table S2: EEG studies data.

**EEG** Reference	QualSyst score (%)[b]	NHMRC[c]	Objectives	Number of subjects analyzed	Age	Female/Male	Tasks and/or conditions (bold: eligible for the review)	Number of trials	Measure	Review conclusions	Locus of interest 10-10 system[a]	Locus of interest Brodmann Areas[a]	Locus of interest Anatomical gyri[a]
**Physiology**													
Huckabee 2003 [12]	17/20 (85%) Strong	IV	**To evaluate the role of the cerebral cortex in the motor planning and initiation of deglutitive behavior, focusing on Bereischaftpotential (BP)**<br><br>**To investigate whether the act of swallowing utilizes cortical motor planning under the condition of volitional swallowing.**	20	Age range: 18-35	10/10	**Task 1: Self-paced breathing 5s pause-preceeded volitional saliva swallowing "with effort"**<br><br>Task 2: Finger press movement	180<br><br>180	Negative potentials: BP onset time and amplitude	Swallowing evokes a 1 phase BP that can be measured on Cz FCz FC1z FC2z during the [-5000ms:0ms] time window<br><br>Trend for lower amplitude for swallowing at 4 time points (p<0.10)<br><br>BP for finger tapping was not significantly earlier than for swallowing<br>No lateralization	Cz<br>FCz<br>FC1z<br>FC2z	BA4<br>BA6<br>BA6L<br>BA6R	Precentral gyrus (M1)<br>PMC and SMA
Satow 2004 [13]	18/20 (90%) Strong	IV	**To clarify whether the hemispheric dominance can be determined in the preparatory period of swallowing or not.**	8	Age range: 24-38	1/7	**Task 1: Self-paced 2-3 ml water swallowing**<br><br>Task 2: Tongue protrusion	240-480<br><br>240-480	Negative potentials: BP amplitude, duration and laterality index	Earlier BP with swallowing (p=0.012), maximum at vertex midline (Cz) during the [-3000ms:0ms] time window.<br>No lateralization.	Cz	BA4	Precentral gyrus (M1)
Hiraoka 2004 [14]	15/22 (68%) Good	IV	**To differentiate among the cortical activities of motor preparation, execution, and regulation of swallowing using Movement Related Cortical potentials (MRCPs)**<br><br>**To document the MRCPs associated with saliva and water swallowing**	7	Age range: 19-37	na	**Task 1: Self-paced volitional saliva swallowing**<br><br>**Task 2: Self-paced volitional water swallowing from glass in right hand (with 10s rest between infusion and swallowing)**	50<br><br>50	Negative potentials BP amplitude and onset time<br><br>-MRCP (negative slow cortical "SMA" potential before uncued voluntary movement)	MRCP/BP amplitude is greater with saliva than water (p=0.035) and can be measured on C3, C4 and CZ within a [-1500ms:0ms] time window.<br>Positive potential amplitude during execution is greater with water than saliva (p=0.048) and can be measured on C3, C4 and CZ within a [0ms:1000ms] time window.<br>No lateralization.	C3<br>C4<br><br>Cz	BA1,3,4L<br>BA1,3R<br><br>BA4	Left Pericentral Cx<br>Right Postcentral gyrus (S1)<br>Precentral gyrus (M1)
Nonaka 2009 [15]	19/22 (86%) Strong	IV	**To compare the waveforms of contingent negative variation (CNV) associated with the command swallowing task with those of movement related cortical potential (MRCP) associated with the volitional (self-paced) swallowing task in healthy adults.**<br><br>To elucidate the effects of human swallowing training on brain activities preceding the onset of swallowing.	10	Mean age: 27.5 (±1.9)	5/5	**Task 1: Self-paced breathing 4-6s pause-preceeded volitional saliva swallowing "with effort"**<br><br>**Task2: Auditory cued Breathing 4s pause-preceeded saliva swallowing task**	>50<br><br>>50	Negative potentials maximum amplitude, duration and area under curve<br>-CNV (negative slow cortical "prefrontal" potential before instructed 2nd stimuli cued motor response)<br>-CNV' (negative slow cortical "prefrontal" potential before the muscle activity after instructed 2nd stimuli cued motor response)<br>-MRCP	Negative preparatory potentials (CNV and MRCP) can be measured on Fz, Cz, Pz, C3, and C4 (mostly Cz and Fz) up to 2sec before the swallowing muscular movement. Their onset time depends on the task type (cued or volitional).<br><br>CNV amplitude stronger than MRCP amplitude (p<0.01)<br><br>Stronger CNV' at Cz (p<0.05)	Fz<br>Cz<br>Pz<br>C3<br>C4	BA6<br>BA4<br>BA7<br>BA1,3,4L<br>BA1,3R	PMC<br>Precentral gyrus (M1)<br>Parietal lobule<br>Left pericentral Cx<br>Right Postcentral gyrus (S1)
Cuellar 2016 [19]	18/22 (82%) Strong	IV	**To use Independant Component Analysis (ICA) to identify bilateral sensorimotor mu components and infrahyoid muscle components in the primarily reflexive pharyngeal and esophageal phases of swallowing** and a voluntary tongue-tapping task<br><br>**To use event-related spectral perturbation (ERSP) to provide measures of sensorimotor activity across time that can be referenced to infrahyoid muscle activity.**<br><br>To validate further use of this non-invasive means of measuring neural responses.	25	Mean age: 29	20/5	**Task 1: Visually cued self-administered 5ml water swallowing**<br><br>Task 2: Tongue tapping	80<br><br>80	Time frequency domain ERS and ERD from Mu rythm	Swallowing execution evokes bilateral Mu ERD rythm localised in BA4 and BA6 with right lateralization and can be mesured in a [0ms:2000ms] time window in α and β bands	C3<br>C4<br>Cz	BA4<br>BA6<br><br>with independent component analysis	Precentral gyrus<br>PMC

Supplementary Table S2 (Continued)

EEG Reference	QualSyst score (%)[b]	NHMRC[c]	Objectives	Number of subjects analyzed	Age	Female/Male	Tasks and/or conditions (bold: eligible for the review)	Number of trials	Measure	Review conclusions	Locus of interest 10-10 system[a]	Locus of interest Brodmann Areas[a]	Locus of interest Anatomical gyri[a]
**Adaptive physiology**													
Jestrović 2014 [16]	20/24 (83%) Strong	IV	**To investigate the stationarity of the EEG signal during swallowing and the effect of sex, age, different brain regions, and the viscosity of the swallowed liquids.**	55	Age range: 18-65	27/28	**Task 1: Self-paced Saliva swallowing**<br>**Task 2: Self-paced water (1cp) swallowing from cup**<br>**Task 3: Self-paced honey (150cP) swallowing from cup**<br>**Task 4: Self-paced nectar (400cP) swallowing from cup**	5<br>5<br>5<br>5	Stationarity (Distance between local and global spectra) and Index of non-stationarity	Whole head EEG shows that swallowing signal is non-stationnary and needs specific methods to be studied.<br>INS increase with viscosity and is the highest with saliva ($p<0.01$).<br>Male participants exhibited higher non-stationarity ($p<0.01$), except for water swallows.	na	na	na
Jestrović 2015 [17]	19/22 (86%) Strong	IV	**To compare the small-world properties of brain networks for swallowing in two head positions: the neutral or natural position, and the chin-tuck head position,**	55	Age range: 18-65	na	**Task 1: Self-paced saliva swallowing neutral position**<br>**Task 2: Self-paced saliva swallowing chin-tuck head position**	5<br>5	Phase synchronization characterized by degree of the Node,<br>Clustering coefficient,<br>Characteristic shortest path length,<br>Local efficiency	Neutral and chin tuck position swallowing networks display small-world characteristics and seems to differ for some features (e.g. clustering coefficient and characteristic path length).<br>Differences are found in α (inhibitory cognitive and motor tasks) and γ bands (performance of cognitive and motor tasks)	na	na	na
Jestrović 2016 [18]	20/22 (91%) Strong	IV	**To compare the brain networks in term of small world properties, according to swallowing of various fluid viscosities, as well as between swallowing in the neutral and chin-tuck head positions**	55	Age range: 18-65<br>Mean age: 38.58 (±14.84)	na	**Task 1: Self- paced water (1cP) swallowing**<br>**Task 2: Self- paced nectar (150cP) swallowing**<br>**Task 3: Self- paced honey (400cP) swallowing**<br>**Task position A: Self- paced neutral position swallowing (with either aforementioned thickness)**<br>**Task position B: Self- paced chin-tuck position swallowing (with either aforementioned thickness)**<br>Every task (1, 2 ,3) was performed in both positions (A and B)	5<br>5<br>5<br>10<br>10	Phase synchronization characterized by degree of the Node,<br>Clustering coefficient,<br>Characteristic shortest path length,<br>Local efficiency	Significant differences in the brain networks in terms of clustering coefficient, characteristic path length and small-worldness depending on the bolus thickness (in α, β, γ, δ, θ bands, $p<0.05$) and the head position (α, β, γ band, $p<0.05$)<br>The functional brain network activated during swallowing has small-world properties.	na	na	na
Jestrović 2018 [21]	19/22 (86%) Strong	IV	**To investigate the effects of external distraction on brain activity during swallowing.**	15	Age range: 18-35	0/15	**Task 1/Condition 1: Self-paced 1ml water swallow without distractor**<br>**Task 1/Condition 2: Self-paced 1ml water swallow with distractor**<br>**Task 2/Condition 1: Self-paced 5ml water swallow without distractor**<br>**Task 2 /Condition 2: Self-paced 5ml water swallow with distractor**<br>**Task 3/Condition 1: Self-paced 10ml water swallow without distractor**<br>**Task 3/Condition 2: Self-paced 10ml water swallow with distractor**	10<br>10<br>10<br>10<br>10<br>10	Phase synchronization characterized by degree of the Node,<br>Clustering coefficient,<br>Characteristic shortest path length,<br>Local efficiency	Significant differences in the brain networks in terms of custering coefficient, characteristic path length and small-worldness depending on the presence of distractors and the swallowed volume (in α, β, γ, δ, θ bands, $p<0.05$)<br>The brain network is different for no-distraction swallowing compared with the brain network constructed during swallowing with distraction<br>These results showed differences in the swallowing of boluses of various volumes in all frequency bands of interest.	na	na	na

Supplementary Table S2 (Continued)

**EEG** Reference	QualSyst score (%)[b]	NHMRC[c]	Objectives	Number of subjects analyzed	Age	Female/Male	Tasks and/or conditions (bold: eligible for the review)	Number of trials	Measure	Review conclusions	Locus of interest 10-10 system[a]	Locus of interest Brodmann Areas[a]	Locus of interest Anatomical gyri[a]
**Patho-physiology**													
Yuan 2017 [20]	22/26 (85%) Strong	III2	**To investigate the effect of tDCS on swallowing apraxia and cortical activation in stroke patients**	9 Stroke patients with swallowing apraxia=3 Healthy subjects=6	Age range: Patients: 48-70 Controls: 45-65	5/4 Patients: 2/1 Controls: 3/3	**Task 1: Auditory cued volitional water swallowing pre-tDCS**<br><br>**Task 2: Transasally provoked water swallowing pre-tDCS**<br><br>Task 1 post tDCS: Auditory cued water volitional swallowing<br><br>Task 2 post tDCS :Transally provoked water swallowing transnasally<br><br>Task 3: Rest pre-tDCS and post-tDCS	20<br><br>20<br><br>20<br><br>20<br><br>2	Signal complexity through Approximate entropy (ApEn)<br><br>ApEn can characterize the changes in brain function by quantifyig and measuring the regularity of the signal.	Regardless of tDCS<br>Physiology: Volitional water swallowing increases ApEn in F4, C4, P3, P4, and T5 ($p<0.01$)<br>Reflexive swallowing increases ApEn in C4 ($p<0.01$)<br><br>Pathology: Volitional water swallowing did not modify ApEn<br>Reflexive swallowing increased ApEn in left sided regions (C3, P3, T5)	P3<br>P4<br>C3<br>C4<br>F4<br>T5<br>T5	BA39L<br>BA39R<br>BA1,3,4L<br>BA1,3R<br>BA9R<br>BA21<br>BA37	Left angular gyrus<br>Right angular gyrus<br>Left pericentral Cx<br>Right postcentral gyrus<br>Right DLPFC<br>Middle temporal gyrus<br>Fusiform gyrus
**Pathology**													
Restrepo 2020 [22]	18/24 (75%) Good	III2	**To determine the activity of the brain cortex of children with Anterior Open Bite (AOB) at rest and during phonation and deglutition**<br><br>To evaluate the association of intelligence quotient (IQ), attention [Test of Variables of Attention (TOVA)], and oxygen saturation with brain activity in subjects with AOB.	14 Patients with AOB=7 Controls=7	Age range: 10-13 Mean age: 11.9	7/7	**Task 1: AOB group-10s self-paced swallowing from glass of water**<br><br>**Task 1: Non-AOB group-10s self-paced swallowing from glass of water**<br><br>Task 2: AOB group-50s phonation task<br><br>Task 2: Non-AOB group-50s phonation task<br><br>Task 3: AOB group- Rest<br><br>Task 3: Non-AOB group- Rest	1<br><br>1<br><br>1<br><br>1<br><br>1<br><br>1	Time-frequency domain: magnitude (Hertz) for each frequency	There was no difference between the two groups for the swallowing execution<br><br>The only difference was found during the rest task between the two groups on C3 and C4 electrodes with a higher left sided activity in the AOB group in α/θ band ($p=0.05$) and on α band ($p=0.02$).	Rest : C3 C4	Rest : BA1,3,4L BA1,3R	Pericentral Cx

AOB: Anterior open bite
BA: Brodmann area
BP: Bereitschaftspotential
CNV: Contingent Negative Variation
Cx: Cortex
MRCP : Movement correlated cortical potential, contains Bereischaftspotential and Negative slope
M1: Primary motor cortex
Na: not available
NF: Neurofeedback
NS: non significative
PFC: Prefrontal cortex
PMC: Premotor cortex
R/L: Right/Left
SE: Standard error
SMA: Supplementary motor area
S1: Primary sensory motor cortex
tDCS: Transcranial Direct-Current Stimulation
±: Standard deviation

Bold text in objectives and tasks reflects the parts included in our review.
a.Correspondance between 10-10 system, Brodman areas and anatomical gyri were performed according to the article data but also Scrivener and Reader 2022 [79], Okamoto 2004 [80] and http://bioimagesuite.com (based on Lacadie et al. 2008 [81]) when coordinates were available.
b. QualSyst score: Quality score from Kmet et al.[11].
c. NHMRC: National Health and Medical Research Council level of evidence. Level I, systematic reviews; level II, randomized control trials; level III-1, pseudo-randomized control trials; level III-2, comparative studies with concurrent controls and allocation not randomized (cohort studies), case control studies, or interrupted time series with a control group; level III-3, comparative studies with historical control, two or more single-arm studies, or interrupted time series without a control group; level IV, case series.

Supplementary Table S3. fNIRS studies data.

**fNIRS** Reference	QualSyst score (%)[b]	NHMRC[c]	Objectives	Number of subjects analyzed	Age	Female/Male	Tasks and/or conditions (bold: eligible for the review)	Number of trials	Measure	Review conclusions	Locus of interest 10-10 system [a]	Locus of interest Brodmann Areas[a]	Locus of interest Anatomical gyri [a]
**Physiology**													
Kober 2014 [44]	20/22 (91%) Strong	IV	**To examine cortical correlates of motor execution and imagery of swallowing using NIRS.**	14 healthy	Mean age: 31.86	7/7	**Task 1: Self-paced volitional 5-6 water swallowing through oral tube**<br><br>Task 2: Self-paced motor imagery of 5-6 swallowing through oral tube	20<br><br>20	OxyHb and DeoxyHb mean relative concentration amplitude	Swallowing activity localised in the bilateral inferior frontal gyri, measured within a [0ms:+25000ms] time window (p<0.05)	T3, T4, FT7, FT8<br>FC5, FC6, FT7, FT8	BA 22<br>BA 44	Inferior frontal gyrus<br>Pars opercularis
Inamoto 2015 [47]	17/22 (77%) Good	IV	**To examine cerebral blood volume dynamics during volitional swallowing using multi-channel fNIRS**<br><br>**To identify the specific regions of the cerebral cortex that exhibited activation.**	15 healthy	Mean age: 26.5 (±1.3)	3/12	**Task: Orally cued 5ml water swallowing**	10	OxyHb relative concentration mean amplitude from [+5s after end of task:+10s]	Using the OxyHb concentration changes, it is possible to visualize the swallowing cortical evoked CBF in the posterior frontal region and its surroundings (p<0.05)	C4, C3<br>C4, C3<br>T4,T3<br>T4,T3<br>F3,F7<br>F8,F7<br>P4,P3	BA4,1<br>BA3<br>BA22<br>BA21<br>BA9,45<br>BA47<br>BA40	Precentral gyrus<br>Postcentral gyrus<br>Superior temporal gyrus<br>Middle temporal gyrus<br>Left middle frontal gyrus<br>Inferior frontal gyrus<br>Supramarginal gyrus
Kamarunas 2018 [50]	23/24 (96%) Strong	IV	**To determine the timing and amplitude characteristics of cortical activation patterns in the right and left precentral motor and postcentral somatosensory regions during spontaneous reflexive saliva swallows using fNIRS.**	14 healthy	Age range: 28-59<br>Mean age: 44	7/7	**Task: Spontaneous swallowing during rest without instruction**	12	OxyHb and DeoxyHb relative concentration Z-score zmplitude, onset time and peak time<br>-Early HDR at [-5:8s]<br>-Late HDR at [8:35s]	In the four region, the mean peak times are situated during the [3-4s] interval (early response) and during the [13-22.5s] interval (late response).<br>Spontaneous uncued swallowing evokes an early cortical response peak during the [0:8s] period. Left S1 response was the earliest at onset (-2s, p<0.008) with stronger responses. This response is non-significantly followed by responses of right M1, right S1 and last left M1.<br>Spontaneous uncued swallowing evokes a late cortical response [8-35s]. Time course across the regions was not significant for the late peak.<br>The strongest HbO2 change were found in left S1 in comparison to left M1 (p<0.005) regions during the early peak<br>The four regions' activity seems independent, as activity correlations were unsufficient with strongest correlations between left S1 and right M1 (*r*=0.63) and both M1 (*r*=0.63)	C4,C3<br><br>C4,C3	BA4 (BA44 in MNI [d])<br>BA1	Precentral gyrus (M1)<br>Postcentral gyrus (S1)
Kober 2018 [51]	21/22 (95%) Strong	IV	**To investigate whether NIRS is sensitive enough to reveal differences in the hemodynamic response over the bilateral IFG between swallowing saliva and water in healthy adults.**<br><br>**To compare the hemodynamic response over the two hemispheres**	16 healthy	Mean age: 23.81 (SE=±0.53)	8/8	**Task 1: Self-paced volitional 5 to 6 water swallowing through oral tube**<br><br>**Task 2: Self-paced volitional 5 to 6 saliva swallowing**	20<br><br>20	OxyHb and DeoxyHb mean relative concentration amplitude and shape	Strongest swallowing evoked response is located bilateraly in the inferior frontal region in pars opercularis (False discovery rate, p<0.10)<br>Differences between water and saliva with higher oxyHb responses for saliva (p<0.05)	FC5, FC6, FT7, FT8<br>F7,F8	BA 44<br>BA 45	Inferior frontal gyrus<br>Pars opercularis
Matsuo 2021 [54]	17/22 (77%) Good	IV	**To investigate the cerebral hemodynamics associated with the MI and ME of a self-feeding activity with chopsticks**	21 healthy	Mean age: 29.4 (±10.2)	"14F and 7F"?	**Task 1: Stopwatch-cued volitional cucumber eating with chopsticks**<br><br>Task 2: Assistant orally-cued motor imagery of cucumber eating ith chopsticks	3<br><br>3	OxyHb mean (5-second moving average with integral mode) relative concentration Z-score amplitude over inferior frontal gyrus	Swallowing execution evokes a typical oxyHb HDR in SMC, PFC and pre-SMA and a oxyHb decrease in SMA and PMA between [5:25s] after onset (p<0.05)	FC3,FC4,FC5,FC6<br>F1,F2,FC1,FC2,FCz<br>C3, C4<br>Fp1,Fp2,F3,F4	BA6 lateral<br>BA6 medial<br>BA1, 4, 3<br>BA9, 10	PMC<br>Pre-SMA, SMA<br>Sensorimotor Cx<br>PFC

Supplementary Table S3 (Continued).

**fNIRS** Reference	QualSyst score (%)[b]	NHMRC[c]	Objectives	Number of subjects analyzed	Age	Female/Male	Tasks and/or conditions (bold: eligible for the review)	Number of trials	Measure	Review conclusions	Locus of interest 10-10 system [a]	Locus of interest Brodmann Areas[a]	Locus of interest Anatomical gyri [a]
**Adaptive physiology**													
Kober 2015 [46]	23/28 (82%) Strong	IV	To address the question whether both hemodynamic parameters of fNIRS, oxy- and deoxy-Hb, can be modulated voluntarily by means of real-time neuro-feedback (NF), when participants imagine swallowing.<br><br>**To study the effects of NIRS-based NF training on swallowing related brain activation patterns, measuring the cortical correlates of ME** and MI of swallowing before and after NF training.	20 healthy	Mean age: 31.86 OxyHb group = 23.8 (SE=±0.47) DeoxyHb group = 25.7 (SE=±1.20)	10/10	**Task 1: Before NF Self-paced volitional 5 to 6 water swallowing through oral tube before NF**<br><br>**Task 1: After NF Self-paced volitional 5 to 6 water swallowing through oral tube**<br><br>Task 2: Before NF - Self-paced motor imagery of 5 to 6 swallowing through oral tube<br><br>Task 2: After NF - Self-paced motor imagery of 5 to 6 swallowing through oral tube	20<br><br>20<br><br>20<br><br>20	OxyHb and DeoxyHb mean relative concentration amplitude	Strongest swallowing evoked response is located bilateraly in the inferior frontal region and measured in a [0ms:+25000ms] time window (p<0.01)<br>This response can be enhanced after deoxyHb Neurofeedback (p<0.05)	FC5, FC6, FT7, FT8<br>T4,T3<br>T3, T4, FT7, FT8<br>F1,FC1,FC3,FC5, FCz	BA 44<br>BA 45<br>BA 22<br>BA 6L	Inferior frontal gyrus
Mulheren 2016 [48]	22/22 (100%) Strong	IV	**To determine whether swallowing function and hemodynamic responses differ in response to different tastes (sour and/or sweet) with mediation by genetic taster status**.<br><br>To study the effect of the presence/absence of a supplemental slow, steady water infusion on both swallowing pace and hemodynamic responses of the primary motor cortex	13 healthy (2 excl.)	Age range: 20-55 Mean age: 29	11/4 (2 excluded)	**Task 1: Self-paced swallowing 3ml bolus medium sour**<br><br>**Task 2: Self-paced swallowing 3ml bolus strong sweet swallowing**<br><br>**Task 3: Self-paced swallowing 3ml bolus deionizes water swallowing**<br><br>**Task 4: Self-paced swallowing 3ml bolus sour+water infusion (0.08 l/min) swallowing**<br><br>**Task 5: Self-paced swallowing 3ml bolus deionized+water infusion (0.08 l/min) swallowing**	30<br><br>30<br><br>30<br><br>30<br><br>30	OxyHb relative concentration Z-score amplitude and timing<br>-"Swallow" HDR at [2-7s]<br>-"Taste" HDR at [17-22s]	Swallowing evoked an early activity peak between [2-7s] in M1, S1 and SMA that is not influenced by the taste (p<0.05)<br><br>Swallowing evoked a late activity [17-22s]influenced by the taste, the highest activity being obtained with sour taste (p<0.05)<br><br>The oxyHb of the bilateral M1, S1 and SMA were similar during the early peak<br><br>During the late peak, oxyHb was significantly greater in M1 and in S1, but was similar in SMA and the dummy region (p<0.05)	C4,C3<br>C4,C3<br>F1,F2,FC1,FC2,FC3, FC4,FT8,FT9,Fz	BA4 (BA44 in MNI [d])<br>BA1<br>BA6	Precentral gyrus (M1)<br>Postcentral gyrus (S1)<br>SMA
Mulheren 2017 [49]	22/24 (92%) Strong	III2	**To study the effects of different cervical vibrations protocols** (different frequencies, either continuous or pulsed) on:<br>-the fundamental frequency of the voice during stimulation in comparison with voicing without stimulation.<br>-the regulation of brain stem control of swallowing through the swallowing frequency.<br>**-the cortical swallowing network on fNIRS recordings during stimulation epochs**<br><br>**To compare the cortical effects of vibratory stimulation during stimulation or between stimulation periods during 20-min stimulation conditions in comparison with sham conditions.**	8 healthy	Mean age: 45.5	5/3	**Condition 1: Spontaneous Swallowing during 10s cervical vibratory stimulation (8 different frequency conditions) 0-30s without instruction**<br><br>**Condition 2: Spontaneous Swallowing after 10s cervical vibratory stimulation (8 different frequency conditions) 30-45s without instruction**<br><br>**Condition 3: Spontaneous Swallowing during sham stimulation 0-30s without instruction**<br><br>Condition 4: Control cortical activity during 10s vibration (regardless of swallowing)	30<br><br>30<br><br>30<br><br>30	OxyHb relative concentration Z-score mean amplitude and timing<br>-Early HDR at [4-7s]<br>-Late HDR at [14-17s]	Early HDR [4:7s] detected in both M1 and S1 and late activity [14:17s]<br>Activity increased in both early and late response with vibrations compared to sham, with varying lateralization p<0.05	C4,C3<br>C4,C3	BA4 (BA44 in MNI [d])<br>BA1	Precentral gyrus (M1)<br>Postcentral gyrus (S1)

Supplementary Table S3 (Continued).

**fNIRS** Reference	QualSyst score (%)[b]	NHMRC[c]	Objectives	Number of subjects analyzed	Age	Female/Male	Tasks and/or conditions (bold: eligible for the review)	Number of trials	Measure	Review conclusions	Locus of interest 10-10 system [a]	Locus of interest Brodmann Areas[a]	Locus of interest Anatomical gyri [a]
**Adaptive physiology**													
Kober 2019 [53]	24/28 (86%) Strong	III2	To compare the trainability of hemodynamic parameters between healthy young and older individuals within one neurofeedback training session.<br>**To investigate if NIRS signal change during executing and imagining swallowing movements is comparable between young and older individuals when no real-time feedback of brain signals is provided.**	43 healthy<br>Young : 24<br>Older : 19	Age range:<br>Young: 21-28<br>Older: 60-84<br>Mean age:<br>Young: 23.5<br>Older: 68	29/14<br>Young group: 17/7<br>Older group: 12/7	**Task 1: Young group - Self-paced volitional 5 to 6 saliva swallowing before NF**<br>**Task 1: Older group - Self-paced volitional 5 to 6 saliva swallowing before NF**<br>Task 2: Young group - Self-paced motor imagery of 5 to 6 saliva swallowing before NF<br>Task 2: Older group - Self-paced motor imagery of 5 to 6 saliva swallowing before NF	10<br>10<br>10<br>10	OxyHb or deoxyHb mean relative concentration amplitude of inferior frontal gyrus	During the swallowing task oxyHb was significantly greater on the left IFG than on the right IFG in the 2 groups ($p<0.05$).<br>No significant difference between young and older subjects (slightly stronger response in youngs)	FC5, FC6, FT7, FT8<br>F7,F8	BA 44<br>BA 45	Inferior frontal gyrus<br>Pars opercularis
**Patho-physiology**													
Lee 2018 [52]	20/22 (91%) Strong	III3	**To investigate prefrontal cortex activity using NIRS, in healthy volunteers and dysphagia patients during swallowing of sweetened/unsweetened and flavored/unflavored jelly**<br>**To determine if taste and flavor stimuli modulate prefrontal cortex function in dysphagia patients.**	12<br>Brain impaired dysph.: 5<br>Controls: 7	Age range:<br>Patients: 66-85<br>Controls: 79-94<br>Mean age:<br>Patients: 75.3 (±6.6)<br>Controls: 85.8 (±6.2)	8/4<br>Patients: 4/1<br>Controls: 4/3	**Task 1: Self-paced swallowing of unflavoured/unsweetened 2ml jelly by straw**<br>**Task 2: Self-paced swallowing of unflavoured/sweetened 2ml jelly by straw**<br>**Task 3: Self-paced swallowing of flavoured/unsweetened 2ml jelly by straw**<br>**Task 4: Self-paced swallowing of flavoured/sweetened 2ml jelly by straw**	3<br>3<br>3<br>3	OxyHb relative concentration Z-score amplitude and timing<br>-Early HDR<br>-Late HDR	In healthy subjects<br>-An early prefrontal oxyHb response to swallowing is measured at about 10s<br>-A late peak is seen at about 26s<br>-Sweetness decreases the responses ($p<0.001$); flavor increases the response ($p<0.001$).<br>No peak in dysphagic subjects.<br>Comparing both groups responses, unsweetened jelly evoked higher responses in controls ($p<0.01$)	Fpz, Fp1,Fp2, F3, F4	BA9, 10	Prefrontal Cx (Superior frontal gyrus, Medial frontal gyrus)
**Pathology**													
Kober 2015 [45]	17/22 (77%) Good	III3	**To use NIRS to examine the cortical correlates of swallowing in patients with dysphagia.**<br>**To compare the brain activation patterns associated with saliva swallowing between dysphagia patients and healthy-matched controls, in terms of time course and topographical distribution of the hemodynamic signal change (oxy-Hb and deoxy-Hb) during swallowing.**<br>To determine the extent to which Motor imagery (MI) and Motor Execution (ME) of swallowing lead to comparable brain activation patterns in stroke patients.	6<br>Brainstem stroke (R and L): 2<br>Cerebral stroke (R and R): 2<br>Gender and age-matched controls: 2	Age range:<br>Patients : 68-80<br>Controls : 64-67	4/2<br>Patients: 3/1<br>Controls: 1/1	**Task 1: Self-paced volitional saliva swallowing 3 times - Controls**<br>**Task 1: Self-paced volitional saliva swallowing 3 times - Cerebral stroke patients**<br>**Task 1: Self-paced volitional saliva swallowing 3 times - Brainstem stroke patients**<br>Task 2: Self-paced motor imagery of saliva swallowing 3 times - Controls<br>Task 2: Self-paced motor imagery of saliva swallowing 3 times - Cerebral stroke patients<br>Task 2: Self-paced motor imagery of saliva swallowing 3 times - Brainstem stroke patients	10<br>10<br>10<br>10<br>10<br>10	OxyHb and DeoxyHb mean relative concentration amplitude and onset time	The strongest swallowing activity is localised in the bilateral inferior frontal gyri ($p<0.1$), measured within a [0ms:+20000ms] time window with peak at 15s<br>Cerebral stroke patients show less activation than controls with later peak ($p<0.1$)<br>Brainstem stroke patients show stronger activation than controls with larger region of activity ($p<0.1$)	FC5, FC6, FT7, FT8<br>F7,F8<br>F7,F8	BA 44<br>BA 45<br>BA 47	Inferior frontal gyrus<br>Pars opercularis

BA: Brodmann area
Cx: Cortex
Dysph. : Dysphagic
Excl. : excluded
M1 : Primary motor cortex
Na: not available
NF : Neurofeedback
NS: non significative
PFC: Prefrontal cortex
PMC : Premotor cortex
R/L: Right/Left
SE: Standard error
SMA : Supplementary motor area
S1 : Primary sensory motor cortex
±: Standard deviation

a.Correspondance between 10-10 system, Brodman areas and anatomical gyri were performed according to the article data but also Scrivener and Reader 2022 [79], Okamoto 2004 [80] and http://bioimagesuite.com (based on Lacadie et al. 2008 [81]) when coordinates were available.
b. QualSyst score: Quality score from Kmet et al.[11].
c. NHMRC: National Health and Medical Research Council level of evidence. Level I, systematic reviews; level II, randomized control trials; level III-1, pseudo-randomized control trials; level III-2, comparative studies with concurrent controls and allocation not randomized (cohort studies), case control studies, or interrupted time series with a control group; level III-3, comparative studies with historical control, two or more single-arm studies, or interrupted time series without a control group; level IV, case series.
d. According to Bioimage Suite based on Lacadie et al. 2008 [81]. Bold text in objectives and tasks reflects the parts included in our review.

Supplementary Table S4. MEG studies data.

**MEG** Reference	QualSyst score(%)[b]	NHMRC[c]	Objectives	Number of subjects analyzed	Age	Female/Male	Tasks and/or conditions (bold: eligible for the review)	Number of trials	Measure	Review conclusions	Locus of interest 10-10 system [a]	Locus of interest Brodmann Areas[a]	Locus of interest Anatomical gyri [a]
**Physiology**													
Loose 2001 [23]	16/22 (73%) Good	IV	**To study the sources of activation evoked by active tongue movement employing MEG**<br><br>**To identify the locality of the major contributors**	5	Age range: 30-49	0/5	**Task 1: Self-paced 5ml water swallowing**<br><br>Task 2: Tongue protraction	100<br><br>100	Time series amplitude, dipole localization	No cortical source found	None	None	None
Abe 2003 [24]	14/22 (64%) Good	IV	**To investigate whether the decision to drink is made just before swallowing, using a 306-channel whole-head neuromagnetometer.**	3	Adults (age not provided)	na	**Task: Self-paced 1mL water bolus by tube**	50	Time series amplitude, onset time and dipole localization	The magnetic dipole during the swallowing preparation is bilaterally located in the cingulate gyrus and SMA and is active between [-1500 and -1100ms] before the volitional swallowing muscular activation.	Na<br>F1,F2,FC1,FC2,FC3, FC4,FT8,FT9,Fz	BA24,32<br>BA6	Cingulate gyrus<br>SMA
Dziewas 2003 [25]	18/22 (82%) Strong	IV	**To study cortical activation during volitional and reflexive water swallowing with whole-head MEG and synthetic aperture magnetometry (SAM)**<br><br>**To compare the cortical representation of swallowing with that of a swallow-related but less complex movement task with an added tongue movement paradigm was included in the study design.**	10	Mean age: 36 (±8.1)	3/7	**Task 1: Self-paced volitional 10ml/min water swallowing by oral tube**<br><br>**Task 2: Provoked 10ml/min water swallowing by transnasal tube with volitional tongue movement**<br><br>Task 3: Tongue propulsion<br><br>Task 4: Resting stage	97±13<br><br>84±4<br><br>97±8<br><br>na	Time-frequency domain ERS and ERD amplitude and lateralization index	The most prominent and consistent activity (α and β ERD) is located in bilateral BA 1,2,3,4,7 in volitional preparation and execution (except only left sided BA7 activity)<br><br>Insula and frontal operculum activity (θ, low γ and high γ ERS) is specifically linked to preparation and execution of swallowing in this experiment<br><br>Pharyngeal reflexive θ band ERS responses are located on left sided over BA9 for preparation and BA7 for execution	C3,C4<br>Cz, C1,C2<br>Pz<br><br>F3,F4<br><br>na<br>F7,F8,FT7,FT8	BA1,2,3<br>BA4<br>BA7<br><br>BA9<br><br>BA13<br>BA44	Postcentral gyrus (S1)<br>Precentral gyrus (M1)<br>Parietal lobule - visuomotor Cx<br>Dorsal Dorsolateral prefrontral Cx<br>Insula<br>Frontal operculum
Watanabe 2004 [27]	16/20 (80%) Strong	IV	**To investigate serial positional changes in the entire activity areas in the cerebral cortex with time until the initiation of swallowing movement.**<br><br>**To define the spatiotemporal relations among regions of the brain involved in the central initiation of human voluntary swallowing using the MEG technique with a larger subject size.**	8	Age range: 25-30	na	**Task 1: Assistant administered cued 3ml water by tube**<br><br>Task 2: Right middle finger extension	30<br><br>30	Equivalent current dipole latency and duration	Swallowing preparation evoked bilateral responses located in ACC, PCC,MFG, IFG and Insula.<br>The mesured sequence is PCC>SMA>ACC>SFG>MFG>IFG>Insula but only PCC and Insula had significant different onset times (p<0,003)<br>Insula and IFG activity where more consistent for swallowing than for finger extension<br>The swallowing activity is measurable during the [-2375ms:-1055ms] time window	na<br>na<br>F7,F8,FT7,FT8<br>na	BA31, 23<br>BA13<br>BA44<br>BA 25, 24, 32, 33	Posterior Cingulate Cx<br>Insula<br>Inferior frontal gyrus<br>Anterior Cingulate Cx
Furlong 2004 [26]	18/22 (82%) Strong	IV	**To use MEG to dissociate the relative cortical contributions of each of the separable components of swallowing in the sensorimotor sequence**<br><br>**To identify the spatio-temporal characteristics of cortical activation during swallowing.**<br><br>**To enhance our appreciation of the relevance of cortical regions to swallowing and provide insight into the mechanisms underlying dysphagia after cerebral injury.**	8	Age range: 26-45	2/6	Task 1: Assistant administered 5ml water in mouth by tube (no swallowiing)<br><br>**Task 2: Cued 5ml water swallowing**<br><br>Task 3: Cued tongue pressure<br><br>Task 4: Resting state	20<br><br>20<br><br>20<br><br>20	Grand average of time-frequency domain ERS and ERD spectral power density	Swallowing execution evokes an activation pattern shifting from caudal pericentral cortex activation to superior postcentral gyri and paracentral lobule<br>In this study, activation (ERD) during swallowing appears more right sided	C3,C4<br>Cz, C1,C2<br>P1,P2,Pz, C3,C4<br><br>C5,C6,P3,P4	BA 3, 1, 2<br>BA4<br>BA5<br><br>BA40	Postcentral gyrus<br>Precentral gyrus<br>Superior parietal lobule - Associated sensory cortex<br>Supramarginal gyrus

Supplementary Table S4 (Continued).

**MEG** Reference	QualSyst score(%)[b]	NHMRC[c]	Objectives	Number of subjects analyzed	Age	Female/Male	Tasks and/or conditions (bold: eligible for the review)	Number of trials	Measure	Review conclusions	Locus of interest 10-10 system [a]	Locus of interest Brodmann Areas[a]	Locus of interest Anatomical gyri [a]
**Physiology**													
Dziewas 2005 [28]	19/22 (86%) Strong	IV	**To apply whole-head MEG in order to study the cortical processing of esophageal sensation in healthy humans**	9	Mean age: 35 (±8.4)	2/7	**Task 1: Self-paced volitional 10ml/min water swallowing by oral tube**<br>Task 2: Direct esophageal stimulation	96±12<br>50	Time-frequency domain ERS and ERD amplitude	During volitional swallowing, β and α activity is left lateralized within the primary sensorimotor cortex.	C3,C4<br>Cz, C1,C2	BA1,2,3<br>BA4	Postcentral gyrus (S1)<br>Precentral gyrus (M1)
Teismann 2009 [32]	20/22 (91%) Strong	IV	**To investigate the temporal characteristics of human swallowing in healthy subjects by means of whole-head MEG and SAM.**	10	Mean age: 35.9<br>Age range: 22-60	2/8	**Task: Self-paced volitional 10ml/min water swallowing by oral tube**	61.2±21.1	Time-frequency domain ERS and ERD spectral power and lateralization index	During the swallowing execution, the primary sensorimotor cortex α and β activity is left sided during [-400:+200ms] then is symetric during [+200ms:+400ms] and last, right sided during [+400:+600ms] (in reference to muscle activation called M1 in the study).	C3,C4<br>Cz, C1,C2	BA1,2,3<br>BA4	Postcentral gyrus (S1)<br>Precentral gyrus (M1)
**Adaptive physiology**													
Teismann 2007 [29]	20/24 (83%) Strong	IV	**To study cortical activity during self-paced volitional swallowing with and without topical oropharyngeal anesthesia with MEG**<br>To evaluate the impact of sensory input in healthy subjects.	10	Mean age: 35.9<br>Age range: 22-60	3/7	**Task 1: Self-paced volitional 10ml/min water swallowing by oral tube**<br>**Task 2: Self-paced volitional 10ml/min water swallowing by oral tube after pharyngeal anesthesia**	60.2±21.5<br>52.7±20.5	Time-frequency domain ERS and ERD spectral power density	During volitional swallowing, β activity is bilateral within the primary sensorimotor cortex and maximum at 300ms. Peripheral sensory suppression reduces the cortical responses, most predominently on the left side (-35%, p<0.05) VS the right side (-28%, p<0.05) without significant lateralization	C3,C4<br>Cz, C1,C2	BA1,2,3<br>BA4	Postcentral gyrus (S1)<br>Precentral gyrus (M1)
Teismann 2009 [31]	21/24 (88%) Strong	IV	**To study cortical activity during self-paced volitional swallowing with and without preceding thermal tactile oral stimulation**	15	Mean age: 30.4<br>Age range: 25-57	8/7	**Condition 1: Self-paced volitional 10ml/min water swallowing by oral tube**<br>**Condition 2: Self-paced volitional 10ml/min water swallowing by oral tube after TTOS**	73.5<br>73.7	Time-frequency domain ERS and ERD spectral power density and lateralization index	In the control condition, the primary sensorimotor cortex α and β activity is stable during [-400:0ms] then is left sided during [0:200ms] and right sided during [200:600ms]. Cold stimulation (TTOS) improves the left α and β activity (p<0.05) during the whole execution sequence with a left lateralisation through [-400ms:+600ms].<br>This suggests the volitional (Oral) phase seems more left sided and the reflexive phase (pharyngo-oesophageal) seems more right sided.	C3,C4<br>Cz, C1,C2	BA1,2,3<br>BA4	Postcentral gyrus (S1)<br>Precentral gyrus (M1)
Teismann 2010 [34]	20/22 (91%) Strong	III3	**To examine with whole-head MEG and compare changes in cortical swallowing processing in young versus elderly subjects**	18<br>Young=9<br>Elders=9	Young<br>Mean age: 3.8<br>Age range: 22-26<br>Older:<br>Mean age: 1.6<br>Age range: 60-85	11/7	**Condition 1: Young volunteers - Self-paced volitional 10ml/min water swallowing by oral tube**<br>**Condition 2: Elder volunteers - Self-paced volitional 10ml/min water swallowing by oral tube**	66.1<br>55.8	Time-frequency domain ERS and ERD spectral power density	In elders, broader and stronger bilateral (p<0.05) activation during preparation and execution in comparison to classical results in young subjects (BA4,3,2,1 α and β ERD bilateral symetrical activity]	C3,C4<br>Cz, C1,C2	BA1,2,3<br>BA4	Postcentral gyrus (S1)<br>Precentral gyrus (M1)

Supplementary Table S4 (Continued).

**MEG** Reference	QualSyst score(%)[b]	NHMRC[c]	Objectives	Number of subjects analyzed	Age	Female/Male	Tasks and/or conditions (bold: eligible for the review)	Number of trials	Measure	Review conclusions	Locus of interest 10-10 system [a]	Locus of interest Brodmann Areas[a]	Locus of interest Anatomical gyri [a]
**Adaptive physiology**													
Suntrup 2013 [38]	27/28 (96%) Strong	III1	To evaluate the effect of tDCS on the swallowing network activity by applying MEG.<br>**To gain insight into the underlying mechanism of action and to link neuroplastic with behavioral changes in swallowing.**	21	Mean age: 26.76 (±3.4)	11/10	**Task 1: Pre-tDCS - Visually cued simple saliva swallowing - "Simple swallow task"**<br>Task 1: Post-tDCS - Visually cued simple saliva swallowing - "Simple swallow task"<br>**Task 2: Pre-tDCS - Visually cued fast saliva swallowing - "Fast swallow task"**<br>Task 2: Post-tDCS - Visually cued fast saliva swallowing - "Fast swallow task"<br>**Task 3: Pre-tDCS -150ms time window-targeted saliva swallowing - "Challenged swallow task"**<br>Task 3: Post-tDCS - 150ms time window-targeted saliva swallowing - "Challenged swallow task"	40<br>40<br>40<br>40<br>40<br>40	Time-frequency domain ERS and ERD spectral power density and lateralization index	In control condition, activity similar to previous reports.<br>The fast swallow task after tDCS increases the pericentral activity in all bands (p=0.006).<br>The challenged task after tDCS increases both pericentral and premotor (PMC and SMA) activity in all bands, and also the parieto-occipital α activity (p=0.007)	C3,C4<br>Cz, C1,C2<br>F1,F2,FC1,FC2,FC3, FC4,FT8,FT9,Fz	BA1,2,3<br>BA4<br>BA6	Postcentral gyrus (S1)<br>Precentral gyrus (M1)<br>PMC and SMA
Suntrup 2015 [40]	26/28 (93%) Strong	III1	**To contribute further knowledge on the cortical topography and frequency–specificity of activation pattern changes during the act of swallowing by taking advantage of MEG.**<br>**To analyze the complete act of swallowing instead using a method allowing to explore the stimulation-induced alterations in the cortical large-scale oscillatory swallowing network beyond the pharyngeal motor cortex.**	14	Mean age: 30.3 (±4.7)	6/8	**Condition 1: Before pharyngeal electrical or sham stimulation - Self-paced volitional 10ml/min water swallowing by oral tube**<br>**Condition 2: Immediately (about 6min) after pharyngeal electrical stimulation - Self-paced volitional 10ml/min water swallowing by oral tube**<br>**Condition 3: Immediately (6min) after sham stimulation - Self-paced volitional 10ml/min water swallowing by oral tube**<br>**Condition 4: 40-55 min after pharyngeal electrical or sham stimulation - Self-paced volitional 10ml/min water swallowing by oral tube**	60 [d]<br>60 [d]<br>60 [d]<br>60 [d]	Time-frequency domain ERS and ERD spectral power density	Control conditions (n°1, 3 and 4) displays similar results to previous reports.<br>Right decrement during PES	C3,C4<br>Cz, C1,C2<br>F1,F2,FC1,FC2,FC3, FC4,FT8,FT9,Fz<br>F3,F4,F7,F8<br>F7,F8,FT7,FT8<br>na<br>C5,C6,CP3,CP4,P3, P4<br>na	BA1,2,3<br>BA4<br>BA6<br>BA9, 10, 45<br>BA44<br>BA13<br>BA40<br>BA43	Postcentral gyrus (S1)<br>Precentral gyrus (M1)<br>PMC and SMA<br>PFC<br>Frontal operculum<br>Insula<br>Lateral parietal cortex (pat.)<br>Subcentral area (pat.)
Muhle 2021 [42]	24/28 (86%) Strong	IV	**To investigate whether anodal tDCS (trancient Direct Current Stimulation) and PES (Pharyngeal Electrical Stimulation) can reverse the effects of experimentally induced pharyngeal hypesthesia on the cortical swallowing network using MEG, using a "virtual lesion model" based on local anesthesia**	10	Mean age: 29.0 (±6.8)	5/5	**Task 1: Baseline post local anesthesia-Self-paced volitional 10ml/min water swallowing by oral tube after local pharyngeal anesthesia**<br>**Task1: A-After tDCS - Self-paced volitional 10ml/min water swallowing by oral tube after local pharyngeal anesthesia**<br>**Task 1: C- After Pharyngeal Electrical Stimulation - Self-paced volitional 10ml/min water swallowing by oral tube after local pharyngeal anesthesia**<br>Task 2: Baseline post local anesthesia-Pneumatic pharyngeal stimulation for 15min through transnasal catheter<br>Task 2: B- After tDCS - Pneumatic pharyngeal stimulation for 15min through transnasal catheter<br>Task 2: D- After PES - Pneumatic pharyngeal stimulation for 15min through transnasal catheter	≈ 70<br>≈ 70<br>≈ 70<br>≈ 400<br>≈ 400<br>≈ 400	Time-frequency domain ERS and ERD spectral power density	After pharyngeal anesthesia, beta, alpha and theta ERD are seen in pericentral cortex with maximum activity in BA6R (p=0.047)<br>PES had a positive treatment effect on cortical activity (p=0.01)whereas tDCS had not. PES might be useful for periferal damage of the swallwoing system, whereas tDCS might be limited to central damage (p>0.05)<br>In their peripheral sensory lesion model of dysphagia, PES as a peripheral stimulation method was able to revert the detrimental effects of reduced sensory input on central swallowing processing, whereas tDCS as a central neuromodulation technique was not. Results may have implications for therapeutic decisions depending on the nature of dysphagia in the clinical context.	C3,C4<br>Cz, C1,C2<br>F1,F2,FC1,FC2,FC3, FC4,FT8,FT9,Fz	BA1,2,3<br>BA4<br>BA6	Postcentral gyrus (S1)<br>Precentral gyrus (M1)<br>PMC and SMA<br>Right BA6 for anesthesia

Supplementary Table S4 (Continued).

**MEG**<br>Reference	QualSyst score(%)[b]	NHMRC[c]	Objectives	Number of subjects analyzed	Age	Female/Male	Tasks and/or conditions (bold: eligible for the review)	Number of trials	Measure	Review conclusions	Locus of interest 10-10 system [a]	Locus of interest Brodmann Areas[a]	Locus of interest Anatomical gyri [a]
**Adaptive physiology**													
Suntrup-Krueger 2021 [43]	20/22 (91%) Strong	IV	**To comprehensively investigate the effect of oral application of a capsaicin-containing red pepper sauce suspension on the biomechanics and neurophysiology of swallowing.**<br><br>To gather further information on the feasibility of capsaicin treatment for dysphagia potential desensitization due to overstimulation was evaluated and the duration and intensity of the effect were assessed by monitoring salivary SP level over time.	10	Mean age: 25.7 (±4.5)	4/6	**Condition 1/Task 1: 5min preconditioning with pure water + 15 min Self-paced 10ml/min pure water swallowing by oral tube**<br><br>**Condition 1/Task 2: 5min preconditioning with pure water + Challenged 10ml/min pure water swallowing by oral tube**<br><br>**Condition 2/Task 1: 5min preconditioning with capsaicinoids + 15 min Self-paced 10ml/min pure water swallowing by oral tube**<br><br>**Condition 2/Task2: 5min preconditioning with capsaicinoids + challenged 10ml/min pure water swallowing by oral tube**<br><br>**Condition 3/Task1: 5min preconditioning with capsaicinoids + 15 min Self-paced 10ml/min capsaicinoids swallowing by oral tube**<br><br>**Condition 3/Task2: 5min preconditioning with capsaicinoids + challenged capsaicinoids 10ml/min swallowing by oral tube**	72±18<br><br>40<br><br>75±30<br><br>40<br><br>71±13<br><br>40	Time-frequency domain ERS and ERD spectral power density	In control condition (Condition 1/Task1), activity was similar to previous reports.<br><br>Challenging conditions (Task 2) increased α and β activity in parieto-occipital cortex<br><br>Capsaicinoids had no effect on cortical MEG but had a direct peripheral effect	C3,C4<br>Cz, C1,C2<br>F1,F2,FC1,FC2,FC3, FC4,FT8,FT9,Fz	BA1,2,3<br>BA4<br>BA6	Postcentral gyrus (S1)<br>Precentral gyrus (M1)<br>PMC and SMA
**Pathology**													
Teismann 2008 [30]	19/22 (86%) Strong	III3	**To study the clinical and neurofunctional changes in swallowing performance and central swallowing processing during remission from botulism intoxication.**	16<br>Botulism patient: 1<br>Controls: 15	Mean age: 27.07<br>Age range: 22-34	7/9	**Condition 1: Self-paced volitional 10ml/min water swallowing by oral tube - 15 healthy subjects**<br><br>**Condition 2: Slow self-paced volitional 10ml/min water swallowing by oral tube - 1 healthy subject**<br><br>**Condition 3: Self-paced volitional 10ml/min water swallowing by oral tube - 1 botulism subject – Day 20**<br><br>**Condition 4: Self-paced volitional 10ml/min water swallowing by oral tube - 1 botulism subject – Day 25**	69.3±21.4<br><br>35<br><br>38<br><br>45	Time-frequency domain ERS and ERD spectral power density	During volitional swallowing, β activity is bilateral within the primary sensorimotor cortex with a max ERD in BA6. γ activity of the insula is linked to the swallowing frequency. Volitional reduction of the swallowing frequency reduce the activity in insula and shift the pericentral activity to the right.<br><br>In the botulism patient, specific β activity of the pericentral cortex disappeared and a specific BA7 activity is observed. However, from a pathophysiological point of view, it is hard to conclude whether the modifications of the cortical activity (loss of activity) are due to Botulism cerebral lesions themselves or to swallowing frequency reduction.	C3,C4<br>Cz, C1,C2<br>F1,F2,FC1,FC2,FC3, FC4,FT8,FT9,Fz<br>Pz<br>na	BA1,2,3<br>BA4<br>BA6<br>BA7<br>BA13	Postcentral gyrus (S1)<br>Precentral gyrus (M1)<br>PMC and SMA<br>(pat.)Posterior parietal cortex<br>Insula
Dziewas 2009 [33]	20/22 (91%) Strong	III3	**To investigate the cortical topography of volitional swallowing in patients with Kenedy disease**	16<br>Kenedy disease patients=8<br>Age-matched controls=8	Patients: Mean age: 53.1 Age range: 43-71<br>Controls: Mean age: 44.6 Age range: 33-60	na	**Condition 1: Kenedy disease - Self-paced volitional 10ml/min water swallowing by oral tube**<br><br>**Condition 2: Healthy controls – Self-paced volitional 10ml/min water swallowing by oral tube**	60 [d]<br><br>60 [d]	Time-frequency domain ERS and ERD amplitude, time and lateralization index	In controls, during swallowing, the β activity of the primary motor cortex is focused in a small area and is left sided during the preparation then more symmetric during execution ($p<0.05$).<br><br>In patients, during swallowing, the β activity of the primary motor cortex is stronger and extended to PFC and posterior parietal cortex and globally right sided during preparation and execution ($p<0.05$)	C3,C4<br>Cz, C1,C2<br>F1,F2,FC1,FC2,FC3, FC4,FT8,FT9,Fz<br>Pz<br>Fpz, Fp1,Fp2, F3, F4	BA1,2,3<br>BA4<br>BA6<br>BA7<br>BA9,10	Postcentral gyrus (S1)<br>Precentral gyrus (M1)<br>PMC and SMA<br>(pat.)Posterior parietal cortex<br>PFC

Supplementary Table S4 (Continued).

MEG Reference	QualSyst score (%)[b]	NHMRC[c]	Objectives	Number of subjects analyzed	Age	Female/Male	Tasks and/or conditions (bold: eligible for the review)	Number of trials	Measure	Review conclusions	Locus of interest 10-10 system [a]	Locus of interest Brodmann Areas[a]	Locus of interest Anatomical gyri [a]
**Pathology**													
Teismann 2011 [35]	21/22 (95%) Strong	III3	**To examine cortical swallowing processing in patients in this early subacute phase after stroke of the cerebrum or the brainstem and focusing on the side of the lesion.**	44<br>Stroke patients=37<br>Age-matched controls=7	Age range: 33-81<br>Patients: Mean age: 63.5(±11.8)<br>Controls: Mean age: 66.3±8.2	14/30<br>Patients : 12/25<br>Controls : 2/5	**Condition 1: Healthy controls – Self-paced volitional 10ml/min water swallowing by oral tube**<br><br>**Condition 2: Hemispheric stroke without dysphagia – Self-paced volitional 10ml/min water swallowing by oral tube**<br><br>**Condition 3: Hemispheric stroke with dysphagia – Self-paced volitional 10ml/min water swallowing by oral tube**<br><br>**Condition 4: Brainstem stroke – Self-paced volitional 10ml/min water swallowing by oral tube**	49.0±12.6<br><br>Right: 42.1±11.6<br>Left: 43.8±18.7<br><br>Right: 45.7±20.3<br>Left: 40.5±19.4<br><br>With dysphagia : 45.8±16.4<br>Without dysphagia : 58.0±15.1	Time-frequency domain ERS and ERD spectral power density	In controls, activation is similar to previous studies with β ERD in [BA4,6 +1,2,3,5,7] (p<0.05)<br><br>In case of hemispheric stroke, higher activity of the DLPFC and insula.<br><br>The presence of dysphagia modifies the results. Hemispheric stroke with dysphagia shows a reduction of ipsilateral pericentral activity with no contralateral activity whereas non dysphagic subject shows bilateral activity similar to controls.<br><br>Brainstem stroke patients shows a right lateralization of their pericentral activity.	C3,C4<br>Cz, C1,C2<br>F1,F2,FC1,FC2,FC3, FC4,FT8,FT9,Fz<br><br>F7, F8, F3, F4	BA1,2,3<br>BA4<br>BA6<br><br>BA45,46,47	Postcentral gyrus (S1)<br>Precentral gyrus (M1)<br>PMC and SMA<br><br>PFC (Pat.)<br><br>Right side for braistem lesions patients
Teismann 2011 [36]	21/22 (95%) Strong	III3	**To study cortical activity during self-paced volitional swallowing on fourteen patients suffering from sporadic ALS with bulbar onset with MEG.**	21<br>ALS Patients=14<br>Controls=7	Patients: Mean age: 58.9(±8.78)<br>Age range: 44-74<br>Controls: Mean age: 57.6(±10.4)<br>Age range: 41-71	5/9	**Condition 1: Healthy controls - Self-paced volitional 10ml/min water swallowing by oral tube**<br><br>**Condition 2: Mildly dysphagic patients - Self-paced volitional 10ml/min water swallowing by oral tube**<br><br>**Condition 3: Severely dysphagic patients - Self-paced volitional 10ml/min water swallowing by oral tube**	58.29<br><br>51.29<br><br>56.86	Time-frequency domain ERS and ERD spectral power density	Healthy controls display similar results to previous reports.<br>In ALS, the more dysphagic, the more right-lateralized is the activity, with a global reduction of the pericentral activity in comparison to controls (p<0.05). No local extension of activity.	C3,C4<br>Cz, C1,C2<br>F1,F2,FC1,FC2,FC3, FC4,FT8,FT9,Fz	BA1,2,3<br>BA4<br>BA6	Postcentral gyrus (S1)<br>Precentral gyrus (M1)<br>PMC and SMA<br><br>Right lateralisation for ALS
Suntrup 2013 [37]	21/22 (95%) Strong	III3	**To evaluate differences in swallow-related cortical activation in dysphagic versus non-dysphagic patients with Parkinson's disease and healthy control subjects using an established swallow paradigm**	30<br>Dysphagic Parkinson patients=10<br>Non-dysphagic Parkinson patients=10<br>Age and gender-matched controls=10	Mean age<br>Patients: 65.5(±12.6)<br>Controls: 66.3(±12.4)	Patient: 9/11<br>Controls: 5/5	**Task: Controls - Self-paced volitional 10ml/min water swallowing by oral tube**<br><br>**Task: Non-dysphagic parkinson - Self-paced volitional 10ml/min water swallowing by oral tube**<br><br>**Task: Dysphagic parkinson - Self-paced volitional 10ml/min water swallowing by oral tube**		Time-frequency domain ERS and ERD spectral power density	In all 3 groups: bilateral pericentral sensorimotor activation<br><br>In patients, a strong decrease in activation was found (p<0.05)<br><br>In non-dysphagic patients: shift of peak activation toward lateral motor, premotor and parietal cortices, reduced and delayed SMA activity (p<0.01)<br><br>In dysphagic patients, reduced activation restricted to the sensorimotor areas (p<0.05).	C3,C4<br>Cz, C1,C2<br>F1,F2,FC1,FC2,FC3, FC4,FT8,FT9,Fz<br>C5,C6,CP3,CP4,P3, P4<br>na	BA1,2,3<br>BA4<br>BA6<br><br>BA40<br>BA43	Postcentral gyrus (S1)<br>Precentral gyrus (M1)<br>PMC and SMA<br><br>Lateral parietal Cx (pat.)<br>Subcentral area (pat.)

Supplementary Table S4 (Continued).

**MEG**<br>Reference	QualSyst score (%)[b]	NHMRC[c]	Objectives	Number of subjects analyzed	Age	Female/Male	Tasks and/or conditions (bold: eligible for the review)	Number of trials	Measure	Review conclusions	Locus of interest 10-10 system [a]	Locus of interest Brodmann Areas[a]	Locus of interest Anatomical gyri [a]
**Pathology**													
Suntrup 2014 [39]	21/22 (95%) Strong	III3	**To investigate cortical swallow-related activation in patients diagnosed with functional dysphagia by means of MEG**<br><br>**To determine whether functional dysphagia is associated with alterations in cortical swallowing processing.**	10<br>Functionnal dysphagia patients=5<br>Controls=5	Mean age Patients : 41.8(±12.5) Controls : 40.2(±16.1)	Patients 2/3 Controls 2/3	**Condition 1: Healthy controls - Self-paced volitional 10ml/min water swallowing by oral tube**<br><br>**Condition 2: Functional dysphagic subjects - Self-paced volitional 10ml/min water swallowing by oral tube**	53±9<br><br>50±24	Time-frequency domain ERS and ERD spectral power density	Healthy controls display similar results to previous reports.<br>In functional dysphagic patients, the pericentral activity isreduced and right lateralised (LI=-0.5050) with specific activity of the right SMA, right insula, right DLPFC and right inferolateral parietal lobe.<br><br>Pericentral activity in healthy subjects is more rostro-medial and in functional dysphagic patient, is more caudo-lateral.<br><br>Right lateralisation in patients	C3,C4<br>Cz, C1,C2<br>F1,F2,FC1,FC2,FC3, FC4,FT8,FT9,Fz<br>F3,F4,F7,F8<br>F7,F8,FT7,FT8<br>na<br>C5,C6,CP3,CP4,P3, P4<br>na	BA1,2,3<br>BA4<br>BA6<br>BA9,45<br>BA44<br>BA13<br>BA40<br>BA43	Postcentral gyrus (S1)<br>Precentral gyrus (M1)<br>PMC and SMA<br>PFC<br>Frontal operculum<br>Insula<br>Lateral parietal Cx (pat.)<br>Subcentral area (pat.)
Suntrup-Krueger 2018 [41]	24/26 (92%) Strong	II	To contribute robust evidence to the value of tDCS in dysphagia rehabilitation and overcome some limitations of previous studies.<br><br>To evaluated the efficacy of a pathophysiologically reasonable tDCS protocol to improve stroke-related oropharyngeal dysphagia, conducting a randomized controlled trial (RCT) in a sufficiently large patient sample with objective clinical outcome measures alongside functional neuroimaging.<br><br>To identify predictors of treatment success, which they hypothesized to be patient-related (age, lesion location/size, stroke and OD severity) and/or treatment-related (timing, tDCS+training vs tDCS alone).	10<br>10 had MEG, over the 59 included patients<br>Sham group=5<br>tDCS group=5	Mean age tDCS: 68.9(±11.5) Controls: 67.2(±14.5)<br>Data from the whole population N=59, no precise data for the MEG groups (N=10))	na (for the MEG group)	**Condition 1: Sham group - Self-paced volitional 10ml/min water swallowing by oral tube**<br><br>**Condition 2: tDCS group - Self-paced volitional 10ml/min water swallowing by oral tube**	60 [d]<br><br>60 [d]	Time-frequency domain ERS and ERD spectral power density	Control conditions (n°1) displays similar results to previous reports.	C3,C4<br>Cz, C1,C2<br>F1,F2,FC1,FC2,FC3, FC4,FT8,FT9,Fz<br>F3,F4,F7,F8<br>na<br>C5,C6,CP3,CP4, CP5,CP6	BA1,2,3<br>BA4<br>BA6<br>BA45<br>BA23,31<br>BA40	Postcentral gyrus (S1)<br>Precentral gyrus (M1)<br>PMC and SMA<br>DLPFC<br>Posterior cingulate Cx<br>Supramarginal gyrus

BA: Brodmann area	M1 : Primary motor cortex	NS: non significative	PMC : Premotor cortex	R/L: Right/Left
Cx : Cortex	Na: not available	Pat.: Results specific to pathological subjects	SE: Standard error	S1 : Primary sensory motor cortex
DLPFC : Dorsolateral PFC	NF : Neurofeedback	PFC: Prefrontal cortex	SMA : Supplementary motor area	±: Standard deviation

a.Correspondance between 10-10 system, Brodman areas and anatomical gyri were performed according to the article data but also Scrivener and Reader 2022 [79], Okamoto 2004 [80] and http://bioimagesuite.com (based on Lacadie et al. 2008 [81]) when coordinates were available.
b. QualSyst score: Quality score from Kmet et al.[11].
c. NHMRC: National Health and Medical Research Council level of evidence. Level I, systematic reviews; level II, randomized control trials; level III-1, pseudo-randomized control trials; level III-2, comparative studies with concurrent controls and allocation not randomized (cohort studies), case control studies, or interrupted time series with a control group; level III-3, comparative studies with historical control, two or more single-arm studies, or interrupted time series without a control group; level IV, case series.
d. Calculated based on a 15min measures with 4 swallowing par min and according to previous similar experiments)
